# Traumatic Tetanus Cured

**Published:** 1831

**Authors:** 


### XLIV.

### TRAUMATIC TETANUS CURED.

A CASE of this kind has recently been detailed by Mr. Miskin, of Horsleydown, where success crowned the efforts of the surgeon. The patient, a man 32

years of age, had his foot punctured by a nail on the 4th of October, and on the 7th there were constitutional symptoms which required the attendance of Mr. Minskin. These symptoms were subdued by purgatives, and every thing appeared to be going on well till the 9th, when trismus was evident. On examination it was found that the wound had assumed a livid colour, with pain shooting up the leg. The wound was freely incised, and dressed with oil of turpentine and laudanum, followed by poultices. Five grains of calomel, two of opium, and five of camphor, were ordered every hour, with a purgative draught at the same time. 10th. Passed a bad night—the medicine every two hours. Mr. Callaway saw him this day, at one o'clock. The patient was more tranquil—the stools black as pitch. Morphine half a grain, with ether and camphor, ordered every three hours. An opiate suppository followed by a turpentine enema. We need not pursue the daily details, but may say that by 17th of the same month, October, the patient was so far recovered as to be able to go into the country for change of air and restoration of health.—*Lancet.*

